# Cigarette Smoke-Induced Gastric Cancer Cell Exosomes Affected the Fate of Surrounding Normal Cells via the Circ0000670/Wnt/β-Catenin Axis

**DOI:** 10.3390/toxics11050465

**Published:** 2023-05-17

**Authors:** Zhaofeng Liang, Shikun Fang, Yue Zhang, Xinyi Zhang, Yumeng Xu, Hui Qian, Hao Geng

**Affiliations:** 1 Jiangsu Key Laboratory of Medical Science and Laboratory Medicine, School of Medicine, Jiangsu University, Zhenjiang 212013, China; 2 Department of Clinical Laboratory, The Affiliated Taizhou People’s Hospital of Nanjing Medical University, Taizhou 225300, China; 3 Department of Urology, Hospital of Anhui Medical University, Hefei 230032, China

**Keywords:** gastric cancer, cigarette smoke, circRNA, exosomes, Wnt/β-catenin

## Abstract

Cigarette smoke is a major risk factor for gastric cancer. Exosomes are an important part of intercellular and intra-organ communication systems and can carry circRNA and other components to play a regulatory role in the occurrence and development of gastric cancer. However, it is unclear whether cigarette smoke can affect exosomes and exosomal circRNA to promote the development of gastric cancer. Exosomes secreted by cancer cells promote cancer development by affecting surrounding normal cells. Herein, we aimed to clarify whether the exosomes secreted by cigarette smoke-induced gastric cancer cells can promote the development of gastric cancer by affecting the surrounding gastric mucosal epithelial cells (GES-1). In the present study, we treated gastric cancer cells with cigarette smoke extract for 4 days and demonstrated that cigarette smoke promotes the stemness and EMT of gastric cancer cells and cigarette smoke-induced exosomes promote stemness gene expression, EMT processes and the proliferation of GES-1 cells. We further found that circ0000670 was up-regulated in tissues of gastric cancer patients with smoking history, cigarette smoke-induced gastric cancer cells and their exosomes. Functional assays showed that circ0000670 knockdown inhibited the promoting effects of cigarette smoke-induced exosomes on the stemness and EMT characteristic of GES-1 cells, whereas its overexpression had the opposite effect. In addition, exosomal circ0000670 was found to promote the development of gastric cancer by regulating the Wnt/β-catenin pathway. Our findings indicated that exosomal circ0000670 promotes cigarette smoke-induced gastric cancer development, which might provide a new basis for the treatment of cigarette smoke-related gastric cancer.

## 1. Introduction

Gastric cancer is the fourth most common malignant cancer and the third most common cause of cancer-related death worldwide, with more than 1 million new cases and 769,000 deaths annually [1]. Many factors are related to the occurrence and development of gastric cancer, including gene mutations, infection, dietary factors, environmental factors and cigarette smoke, among other [2,3]. Increasing evidence has shown that cigarette smoke is closely related to the initiation and progression of gastric cancer [2]. Our previous studies also found that cigarette smoke exposure induced malignant transformation and epithelial–mesenchymal transition (EMT) in mouse gastric tissues [4,5]. Although progress has been made in understanding the relationship between cigarette smoke and gastric cancer, the underlying mechanisms are still unclear. Whether cigarette smoke-induced gastric cancer cells can somehow affect surrounding normal cells and promote the development of gastric cancer is also not clear.

Exosomes are an important component of extracellular vesicles, with a size of 30–150 nm, and they are secreted by almost all cell types. Exosomes are an important part of the communication system between cells and within organs, and they can transfer active molecules and biological signals from one cell type or tissue to another [6,7]. Many studies have indicated that exosomes play a decisive role in the occurrence and development of gastric cancer [8,9]. The results of Yoon et al. identified that gastric cancer exosomes induce the transformation and field cancerization of the surrounding gastric epithelial cells [10]. Another study showed that cancer-associated fibroblasts secrete exosomal miR-522 to inhibit ferroptosis in gastric cancer cells by targeting ALOX15 and blocking lipid reactive oxygen species (ROS) accumulation [11]. Cao et al. demonstrated that linc00852 from cisplatin-resistant gastric cancer cell-derived exosomes regulates the COMM domain protein 7 to promote the resistance of recipient cells [12]. It was further reported that exosomes inhibit HSP90 degradation and promote gastric cancer progression by regulating the circSHKBP1/miR-582-3p/HUR axis [13]. Zhang et al. found that circNRIP1 affects metabolic changes in gastric cancer cells and promotes the occurrence and development of gastric cancer through exosome transport and the regulation of the miR-149-5p-AKT1/mTOR axis [14]. Previous studies by our research team also found that exosomes transport active molecules and play a regulatory role in the occurrence and development of gastric cancer [15,16,17]. However, it is unclear whether cigarette smoke can affect exosomes and their transport molecules, such as circular RNAs (circRNAs), to promote the occurrence and development of gastric cancer.

CircRNAs are covalently closed noncoding RNA with obvious tissue specificity and cell specificity [18,19]. The abnormal expression of circRNAs is closely related to the occurrence and development of gastric cancer. Previous results have shown that METTL14-mediated circORC5 mA modification inhibited the progression of gastric cancer by regulating the miR-30c-2-3p/AKT1S1 axis [20]. Furthermore, a study by Peng et al. revealed that circAXIN1 encodes protein AXIN1-295aa, which activates the Wnt/β-catenin pathway to promote gastric cancer progression [21]. Moreover, as the protein decoy of IGF2BP3, circTNPO3 regulates the Myc/Snail axis to inhibit gastric cancer cell proliferation and metastasis [22]. A previous study by our team found that circDIDO1 encodes DIDO1-529aa and inhibits gastric cancer development by regulating PRDX2 stability [15].

CircRNA transport through exosomes has an important role in the occurrence and development of gastric cancer [23]. Exosomal cirHKBP1 promotes the progression of gastric cancer by regulating the miR-582-3p/HuR/VEGF axis and inhibiting Hsp90 degradation [13]. Exosomal circNEK9 accelerates the progression of gastric cancer by regulating the miR-409-3p/MAP7 axis [24]. Li et al. reported that the exosome-mediated transport of circ29 promotes angiogenesis and gastric cancer progression by targeting the miR-29a/VEGF pathway [25]. Studies have also found that cigarette smoke promotes the occurrence and development of diseases by affecting the secretion or transport of active components of exosomes [26,27,28,29]. In addition, cigarette smoke can regulate the expression of circRNAs [30,31,32,33]. However, it is unclear whether cigarette smoke can regulate the expression of exosomal circRNA derived from gastric cancer cells, ultimately affecting the surrounding normal cells to promote the development of gastric cancer.

In summary, we speculated that circRNAs derived from cigarette smoke-induced gastric cancer cell exosomes might affect surrounding normal cells to promote gastric cancer development. As expected, we present in vitro evidence to demonstrate that cigarette smoke-induced gastric cancer cell exosomes can enter surrounding normal cells to promote the development of gastric cancer. Moreover, we found that circ0000670 is a key exosomal component that promotes the development of cigarette smoke-related gastric cancer via the Wnt/β-catenin pathway.

## 2. Materials and Methods

### 2.1. Cell and Cell Culture

The human gastric cancer cell lines HGC-27 and AGS were purchased from Shanghai EK-Bioscience Biotechnology (Shanghai, China). Human gastric epithelial cells (GES-1) were purchased from Shanghai GEFAN Biotechnology (Shanghai, China). HGC-27 cells were cultured in RPMI-1640 medium (BioInd biological industries, Israel, catalog number: 01-100-1A). GES-1 and AGS cells were cultured in RPMI-1640 medium (BioInd biological industries, Kibbutz Beit Haemek, Israel). All cells were cultured in medium supplemented with 10% FBS (Bovogen Biologicals, Keilor East, Australia, catalog number: SFBS) at 37 °C and 5% CO_2_.

### 2.2. Preparation of Cigarette Smoke Extract

Cigarette smoke extract was prepared daily before use according to the reported method [34]. Briefly, one filterless 3R4F reference cigarette (9.4 mg tar and 0.73 mg nicotine/cigarette) was lit and the smoke was continuously drawn through a glass syringe containing 10 mL of fetal bovine serum-free medium pre-warmed to 37 °C at a rate of 5 min/cigarette to generate a cigarette smoke extract solution. A control solution was prepared with the same protocol, except that the cigarette was unlit. The resulting solution was adjusted to pH 7.4 and then filtered through a 0.22 μm pore filter. The obtained suspension was referred to as a 100% cigarette smoke extract solution.

### 2.3. Cell Counting Kit-8 Assay

For the cell counting kit-8 (CCK8) assay, which can be used to detect changes in cell viability, cells were digested, resuspended, counted and planted in 96-well plates (1 × 10^3^ cells/well) [34] (Corning, New York, NY, USA, catalog number: 3599). Next, 110 µL medium containing 10 µL CCK-8 reagent (Vazyme, Nanjing, China, catalog number: A311-02) was added to each well and then incubated for 2 h at 37 °C. The absorbance of each well at 450 nm was measured using a microplate reader (Themo, Waltham, MA, USA). The experiment was repeated at least three times in each group.

### 2.4. Colony Formation Assay

Cells were digested, resuspended, counted and seeded into 6-well plates (1000 cells/well) for the indicated time [35]. The culture medium of cells in each group was changed every 3 days. At the end of the incubation period, the cells were fixed with 4% paraformaldehyde and stained with crystal violet.

### 2.5. Cell Migration Assays

The exponential growth period cells (4 × 10^4^) were seeded into the upper chamber in 24-well plates. The lower chamber was filled with 500 µL medium with 10% FBS. After 12–24 h of culture, these migrated cells at the bottom of the transwells (8 μm) were fixed with 4% paraformaldehyde, stained with crystal violet and then photographed under a microscope [15].

### 2.6. Lentiviral Transfection

The circ0000670 overexpression, knockdown and control lentivectors were purchased from Hanbio Biotechnology (Shanghai, China). Cells in good condition at the logarithmic growth stage were seeded in 6-well plates (8 × 10⁴ cells/well). After 24 h, lentivirus particles at an appropriate MOI (when overexpressed, the MOI of HGC-27 cells is 125, while that of AGS cells is 60. For knockdown, the MOI of HGC-27 cells is 100, while that of AGS cells is 60) were then added to the medium and incubated for 24–48 h. An appropriate concentration of puromycin and 10% FBS medium were used to select the stably transfected cell lines [35].

### 2.7. RNA Extraction and Real-Time PCR

Total RNA was isolated from cells using Trizol (Gibco, New York, USA, catalog number: 15596018) and other reagents according to the experimental protocol. Reverse transcription was performed according to the protocol of the reverse transcription kit manufacturer (Vazyme, Nanjing, China, catalog number: R111-02) using 1 μg RNA [36]. A real-time PCR experiment was carried out on a Step One Plus Real Time PCR System (ABI, Shrewsbury, MA, USA) by using AceQ qpcr Sybr green master mix (Vazyme, Nanjing, China, catalog number: Q111). β-actin served as the loading control. Fold changes in genes expression were evaluated using the 2^−ΔΔ*C*t^ method. The primers were synthesized by biological companies (Invitrogen, Waltham, MA, USA) and the primer sequences are listed in Table 1.

### 2.8. Agarose Gel Electrophoresis

We laced the agarose gel (2.0%) in the electrophoresis tank, mixed 10 μL of PCR product, buffer solution and marker, then added this into the sample well. We performed electrophoresis for 40 min at 110 V then EB staining for 10 min, and the results were observed with a gel imaging system [37].

### 2.9. Western Blotting

Total proteins from cells were harvested and lysed in radioimmunoprecipitation buffer supplemented with 1% protease inhibitors (Pierce, Dorchester, MA, USA, catalog number: 88668). Equal amounts of the total protein were separated on 7.5%–10% SDS-polyacrylamide gel and transferred to 0.45 μm PVDF membranes [38] (Millipore, Burlington, MA, USA, catalog number: IPVH00010). After being blocked with 5% skimmed milk for 1 h, the membranes were incubated with the primary antibodies for GAPDH (1:5000, Bioworld, Minneapolis, MN, USA, catalog number: AP0063), Vimentin, SOX2 (1:1000, Bioworld, Minneapolis, MN, USA, catalog number: BS1491, MB0064), E-cadherin, N-cadherin (1:1000, CST, USA, catalog number: 3195T, 13116P), NANOG, LIN28 and CD44 (1:500, SAB, Los Angeles, CA, USA, catalog number: 21423, 21626) at 4 °C overnight. After being washed three times with tris-buffered saline and Tween, the membrane was incubated with horseradish peroxidase-conjugated secondary antibody (1:5000, Bioworld, Minneapolis, MN, USA) for 1 h. Then, the protein bands were visualized using an enhanced chemiluminescent substrate detection system (Millipore, Burlington, MA, USA, catalog number: WBKLS0500).

### 2.10. Extraction and Identification of Exosomes

We inoculated the same number of cells into the culture dishes, and then each group of cells received different treatments. We collected cell culture supernatant and extracted exosomes from each group of cells using the ultracentrifugation method as reported previously [39]. Transmission electron microscopy was used to detect the morphology of exosomes, a NanoSight nanoparticle tracking analyzer was used to detect the particle size of exosomes and Western blotting was used to detect the expression of markers such as CD9, CD63, CD81, Albumin, Calnexin, HSP70, etc.

### 2.11. NanoSight Nanoparticle Tracking

Exosomes of gastric cancer cells were diluted to the appropriate proportion with PBS (1:1000–1:5000); then, 1 mL of the suspension was analyzed with a NanoSight nanoparticle analyzer [40] (Particle Metrix, Dusseldorf, Germany). Changes in the particle size and concentration of exosomes were analyzed using NanoSight nanoparticle tracking.

### 2.12. Transmission Electron Microscope Scanning

The gastric cancer cell exosome suspensions, with an appropriate dilution of PBS, were transferred to copper mesh and allowed to stand at room temperature for 2–5 min, and then counterstained with 3% phosphotungstic acid for 1–3 min. The morphology of gastric cancer cell exosomes was observed with a transmission electron microscope [41] (Philips, Amsterdam, The Netherlands).

### 2.13. Immunofluorescence Assay

Exosomes were added to DIL cell tracer at a ratio of 1:1000, mixed well and placed in a 37 °C constant-temperature incubator for 30 min. Cells were centrifuged at 4 °C at 1500× *g* for 30 min and resuspended in PBS. Cells were fixed with 4% paraformaldehyde for 20 min and washed with PBS. They were then treated with 0.2% triton X-100 for 10 min and washed with PBS. Serum was used for blocking for 30 min, and samples were washed three times with PBS. A β-actin antibody was incubated with the cells overnight at 4 °C, and they were then washed three times with PBS. The secondary antibody was incubated with the sample at room temperature for 2 h. DAPI staining and PBS washing were then performed three times. Cells were observed under a fluorescence microscope.

### 2.14. Ethics Statement

The gastric cancer tissues and adjacent noncancerous tissues were collected from advanced gastric cancer patients with a history of smoking for more than 5 years who had not received chemotherapy at the affiliated hospital of Jiangsu University. This study was approved by the institutional ethical committee of Jiangsu University (2012258) and written informed consent was obtained from all patients prior to tissue collection.

### 2.15. Statistical Analysis

All the statistical data are presented as mean ± standard deviation and were analyzed by using SPSS software 22.0 (SPSS, Chicago, IL, USA). Unpaired Student’s *t*-test and one-way analysis were used according to actual conditions. Values of *p <* 0.05 were considered significant.

## 3. Results

Cigarette smoke extract promotes EMT and stemness gene expression in gastric cancer cells.

**Figure 1:**
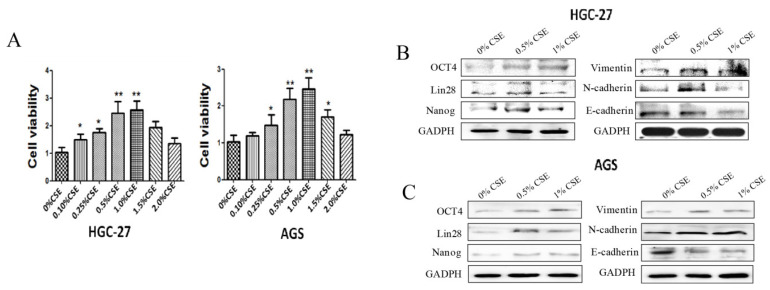
Cigarette smoke promotes EMT and stemness of gastric cancer cells. Gastric cancer cells (HGC-27 and AGS) were treated with cigarette smoke extract for 4 days. (**A**) Effects of different concentrations of cigarette smoke extract on the activity of gastric cancer cells. (**B**,**C**) Protein expression of stemness and EMT markers in gastric cancer cells after cigarette smoke extract exposure. * *p* < 0.05, ** *p* < 0.01.

To investigate the effect of cigarette smoke on gastric cancer cells, gastric cancer cells (HGC-27 and AGS) were treated with various concentrations of cigarette smoke extract for 4 days, and 0.5% cigarette smoke extract was selected for the following experiments (Figure 1A). We found that cigarette smoke extract promoted the expression of OCT4, Lin28, Nanog, Vimentin and N-cadherin (Figure 1B,C). At the same time, the expression of E-cadherin was inhibited (Figure 1B,C). These results suggested that cigarette smoke could promote the EMT and stemness of gastric cancer cells.

### 3.1. Effect of Cigarette Smoke on Exosomes of Gastric Cancer Cells

**Figure 2:**
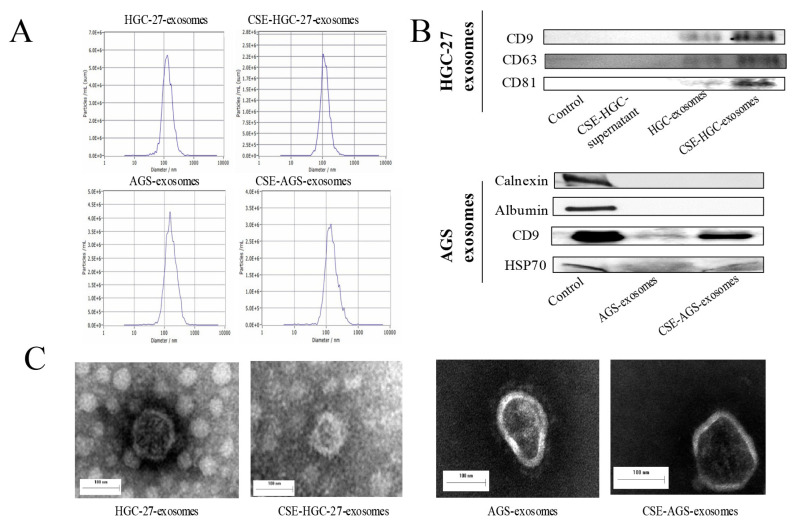
Effect of cigarette smoke on exosomes of gastric cancer cells. Gastric cancer cell exosomes were extracted before and after cigarette smoke exposure. (**A**) Detection of exosomes with a nanoparticle tracking analyzer. (**B**) Exosome-labeled proteins such as CD9, CD63, CD81, Calnexin, Albumin and HSP70 were detected using Western blotting. (**C**) The morphology of exosomes before and after cigarette smoke extract treatment were observed using transmission electron microscopy.

We next extracted the exosomes secreted by gastric cancer cells before and after cigarette smoke extract exposure and analyzed the effect of cigarette smoke on the exosomes of gastric cancer cells. We found that compared with the group without cigarette smoke extract exposure, cigarette smoke extract did not significantly affect the particle size or average secretion level of exosomes (Figure 2A). We identified the extracted exosomes using surface marker proteins (Figure 2B). Transmission electron microscopy showed that exosomes were spherical or oval vesicles, and there were no significant changes in the morphology before and after cigarette smoke extract exposure (Figure 2C). These results suggest that cigarette smoke had no significant effect on the particle size, secretion or surface markers of exosomes secreted by gastric cancer cells.

### 3.2. Cigarette Smoke-Induced Exosomes Promote the Stemness and EMT of GES-1 Cells

**Figure 3:**
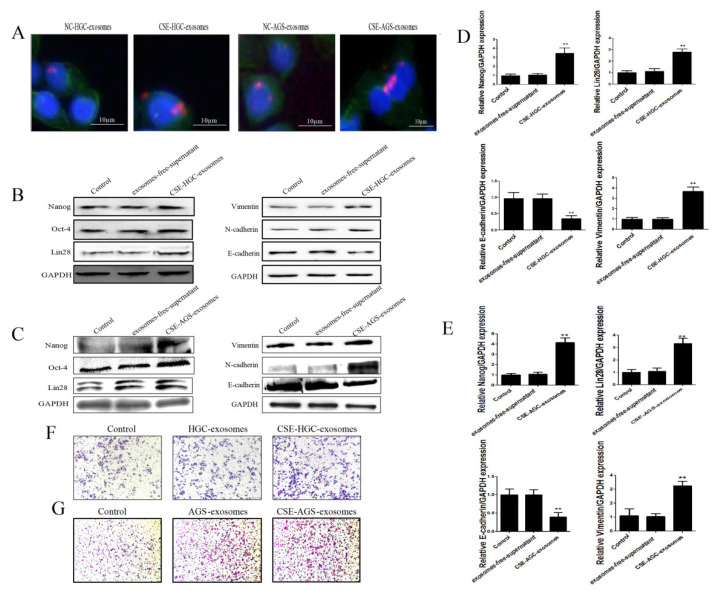
Cigarette smoke-induced exosomes promote the stemness and EMT of GES-1 cells. Cigarette smoke-induced GC cell exosomes were administered to GES-1 cells for 4 days. (**A**) Exosomes of gastric cancer cells were taken up by GES-1 cells. (**B**,**C**) Effect of cigarette smoke-induced exosomes on the protein levels of stemness and EMT markers. (**D**,**E**) Effect of cigarette smoke-induced exosomes on stemness and EMT marker mRNA expression. (**F**,**G**) Cigarette smoke-induced exosomes promoted GES-1 cell migration. ** *p* < 0.01.

The enhancement of the stemness and EMT characteristic of cells plays an important role in the occurrence and development of gastric cancer. To clarify whether cigarette smoke-induced gastric cancer cell exosomes could affect surrounding normal cells to promote the development of gastric cancer, 300 μg of gastric cancer cell exosomes treated with cigarette smoke extract was cocultured with GES-1 cells. An immunofluorescence assay showed that the exosomes treated with cigarette smoke extract were absorbed by GES-1 cells (Figure 3A). Changes in the protein and mRNA levels of stemness and EMT makers in GES-1 cells before and after treatment with cigarette smoke-induced exosomes were detected (Figure 3B–E). The results showed that cigarette smoke-treated exosomes from gastric cancer cells promoted the expression of Vimentin and N-cadherin and inhibited the expression of E-cadherin. Furthermore, the migration ability of GES-1 cells was significantly enhanced (Figure 3F,G). In addition, cigarette smoke-treated exosomes increased the expression of stemness genes (Nanog, OCT4, Lin28). These data revealed that cigarette smoke-treated exosomes of gastric cancer cells promote the stemness and EMT of GES-1 cells, and might play an important role in gastric cancer progression.

### 3.3. Circ0000670 Was Highly Expressed in Cigarette Smoke-Exposed Gastric Cancer Cells and Exosomes

**Figure 4:**
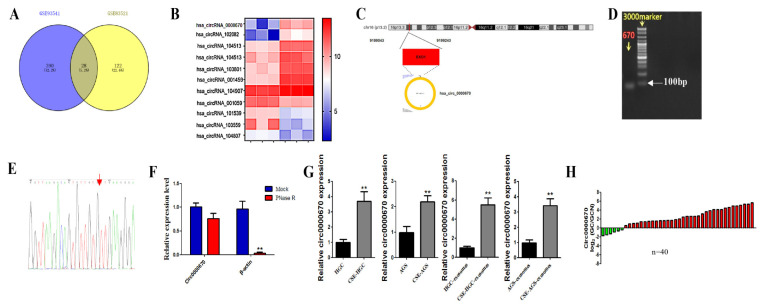
Circ0000670 is highly expressed in cigarette smoke-exposed gastric cancer cells and exosomes. The differentially expressed circRNAs were screened and verified in gastric cancer tissues, gastric cancer cells and exosomes. (**A**) Screening of differentially expressed circRNAs from the GEO database. (**B**) Major circRNAs with obvious differential expression. (**C**) Schematic diagram of mode of circ0000670 formation. (**D**) The results of agarose gel electrophoresis. (**E**) Sanger sequencing results of PCR products of circ0000670. (**F**) The level of circ0000670 in GSE-1 cells after RNase R treatment compared with the level of β-actin. (**G**) Expression of circ0000670 in gastric cancer cells and their exosomes exposed to cigarette smoke. (**H**) Expression of circ0000670 in the tissues of 40 gastric cancer patients with a smoking history. ** *p* < 0.01, compared with control.

Emerging evidence indicates that circRNA plays an important role in the progression of gastric cancer. To determine whether circRNA is involved in the process through which cigarette smoke-induced exosomes promote gastric cancer progression, we analyzed circRNA microarray data (GSE83521 and GSE93541) from the Gene Expression Omnibus database (GEO, https://www.ncbi.nlm.nih.gov/geo/, accessed on 31 December 2021) to obtain circRNA expression profiles, which were verified in the tissues of gastric cancer patients with a smoking history (Figure 4A,B). The results of agarose gel electrophoresis (Figure 4D) and Sanger sequencing (Figure 4E) showed that the circ0000670 primer design was successful. The results showed that circ0000670 expression was significantly up-regulated in gastric cancer tissues. We also found that cigarette smoke could up-regulate the expression of circ0000670 in gastric cancer cells and the exosomes secreted by gastric cancer cells (Figure 4G). In addition, we found that the expression of circ0000670 showed an increasing trend in gastric cancer patients with a smoking history (*n* = 40, 34/40) (Figure 4H). These results determined that circ0000670 was highly expressed in cigarette smoke-exposed gastric cancer cells and exosomes.

### 3.4. Role of Circ0000670 in Cigarette Smoke-Induced Exosomes Promoted GES-1 Cell Stemness and EMT

**Figure 5:**
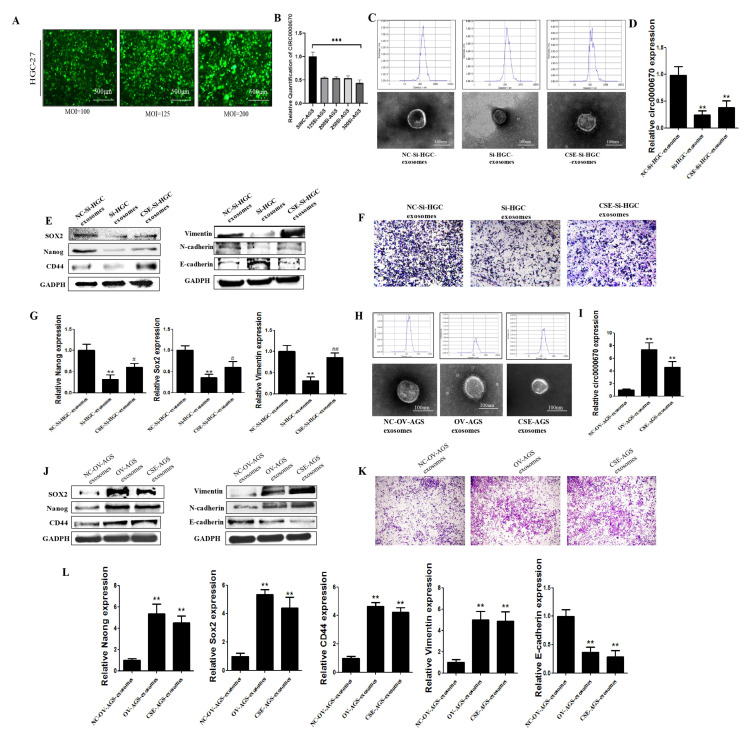
Role of circ0000670 in the promotion of GES-1 cell stemness and EMT by cigarette smoke-induced exosomes. A circ0000670 knockdown lentivirus vector was transferred into the cigarette smoke-induced gastric cancer cells, and exosomes were extracted to treat GES-1 cells. (**A**) Cell infection after treatment with circ0000670 knockdown lentivirus vector. (**B**) Expression of circ0000670 in gastric cancer cells after knockdown. (**C**) Effects of circ0000670 knockdown on the morphology and particle size of HGC-27 exosomes. (**D**) Expression of circ0000670 in exosomes after circ0000670 knockdown. (**E**) Protein expression of stemness and EMT markers after knockdown. (**F**) Changes in migration ability of GES-1 cells after knockdown of circ0000670. (**G**) mRNA expression of stemness and EMT markers after knockdown. (**H**) Effects of overexpression of circ0000670 on the morphology and particle size of AGC exosomes. (**I**) Expression of circ0000670 in exosomes after circ0000670 overexpression. (**J**) Protein expression of stemness and EMT markers after circ0000670 overexpression. (**K**) Changes in migration ability of GES-1 cells after overexpression of circ0000670. (**L**) mRNA expression of stemness and EMT markers after overexpression. ** *p* < 0.01, *** *p* < 0.001, compared with control, ^#^ *p* < 0.05, ^##^ *p* < 0.01 compared with si-HGC-exosomes.

To study the mechanism through which circ0000670 from CS-treated exosomes promotes the stemness and EMT of GES-1 cells, we constructed circ0000670 knockdown and overexpression lentivirus vectors. Cigarette smoke-induced gastric cancer cells were transfected with the circ0000670 knockdown and overexpression lentivirus vectors, and the expression of circ0000670 in exosomes was detected. The knockdown lentivirus had no significant effect on the particle size and morphology of exosomes (Figure 5C). Our results further showed that the circ0000670 knockdown lentivirus vector could significantly inhibit the high expression of circ0000670 in cigarette smoke-treated exosomes (Figure 5D). The promoting effect of cigarette smoke-treated exosomes on the protein and mRNA expression of stemness and EMT markers of GES-1 cells was significantly inhibited by the low expression of circ0000670 (Figure 5E,G). The migration ability of GES-1 cells was significantly weakened (Figure 5F). Moreover, the circ0000670 overexpression lentivirus vector increased the expression of circ0000670 in gastric cancer cell exosomes (Figure 5I). The results suggested that the overexpression of circ0000670 in exosomes significantly promotes the protein and mRNA levels of stemness and EMT markers of GES-1 cells (Figure 5J,L). The migration ability of GES-1 cells was also enhanced (Figure 5K). These results indicated that circ0000670 plays a crucial role in the development of cigarette smoke-induced gastric cancer.

The Wnt/β-catenin pathway might be a downstream effector of the cigarette smoke-induced exosomal circ0000670-mediated promotion of gastric cancer progression.

To further explore the mechanism through which cigarette smoke-induced exosomal circ0000670 promotes the development of gastric cancer, we performed bioinformatics analysis and found that circ0000670 was associated with the Wnt/β-catenin signaling pathway. Gastric cancer cells were treated with the β-catenin-targeting inhibitor jw55, cigarette smoke and lentivirus, alone or in combination, and then the exosomes were extracted.

**Figure 6:**
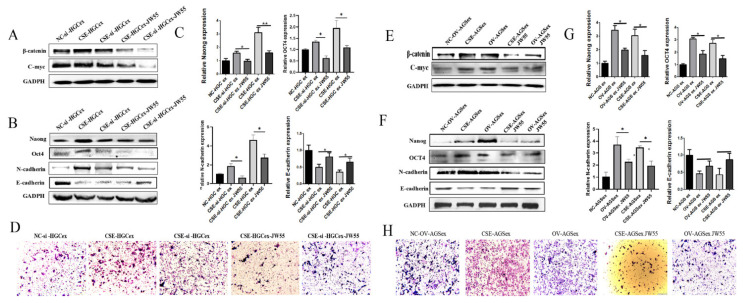
Wnt/β-catenin pathway regulates the gastric cancer-promoting effect of cigarette smoke-induced exosomes. A circ0000670 overexpression lentivirus and the β-catenin-targeting inhibitor jw55 were used to treat cigarette smoke-induced GC cells alone or in combination, and exosomes were extracted to treat GES-1 cells. (**A**) Changes in the protein levels of β-catenin and c-Myc. (**B**) Changes in the protein levels of stemness and EMT markers in GES-1 cells. (**C**) Changes in the mRNA levels of stemness and EMT markers in GES-1 cells. (**D**) Changes in the migration ability of GES-1 cells. (**E**) Changes in the protein levels of β-catenin and c-Myc. (**F**) Changes in the protein levels of stemness and EMT markers in GES-1 cells. (**G**) Changes in the mRNA levels of stemness and EMT markers in GES-1 cells. (**H**) Changes in the migration ability of GES-1 cells. * *p* < 0.05, ** *p* < 0.01, compared with control.

The results showed that the high expression of β-catenin and c-Myc induced by cigarette smoke was inhibited by jw55 in gastric cancer cells. Moreover, circ0000670 overexpression significantly up-regulated the expression of β-catenin and c-Myc in gastric cancer cells, whereas jw55 inhibited this (Figure 6A). The expression of β-catenin, c-Myc and stemness genes as well as EMT, which was promoted by gastric cancer cell exosomes treated with cigarette smoke, was blocked in the jw55 group (Figure 6A–C). Gastric cancer cell exosomes of the circ0000670overexpression group up-regulated the expression of β-catenin, c-Myc and stemness genes and promoted EMT in GES-1 cells. Exosomes from GC cells of the combined jw55-treated and circ0000670 overexpression group inhibited the enhanced expression of stemness genes, β-catenin, c-Myc and EMT caused by circ0000670 overexpression.

It was also found that inhibition of the Wnt/β-catenin pathway could inhibit the expression of stemness genes and EMT in gastric cancer cells (Figure 6E–H). Furthermore, the circ0000670 knockdown lentivirus vector inhibited the up-regulation of β-catenin and c-Myc expression induced by cigarette smoke (Figure 6E). The circ0000670 knockdown virus combined with jw55 treatment significantly inhibited the cigarette smoke-induced up-regulation of β-catenin and c-Myc expression compared to that with the jw55 treatment alone. Further study found that the exosomes of the jw55 treatment group of gastric cancer cells inhibited the expression of stemness genes and EMT in GES-1 cells. Meanwhile, exosomes of gastric cancer cells in the circ0000670 knockdown group inhibited the up-regulation of β-catenin and c-Myc expression induced by cigarette smoke. Exosomes of gastric cancer cells treated with the circ0000670 knockdown vector and jw55 significantly inhibited the cigarette smoke-induced up-regulation of β-catenin, c-Myc, stemness gene expression and EMT compared to those with treatment alone (Figure 6E–H). Based on these results, we speculated that the Wnt/β-catenin pathway might be downstream of cigarette smoke-induced exosomal circ0000670 with respect to the promotion of gastric cancer progression.

## 4. Discussion

There is substantive evidence demonstrating that cigarette smoke is one of the primary causes of gastric cancer. However, the molecular mechanisms by which cigarette smoke promotes gastric cancer are not well established. Exosomes transmit active substances and signals to surrounding cells, which promote the development of gastric cancer. In this study, we found that cigarette smoke enhances the proliferation, stemness and EMT of gastric cancer cells. Furthermore, exosomes derived from cigarette smoke-treated gastric cancer cells promoted the stemness and EMT process of GES-1 cells. In addition, our data indicated that exosomal circ0000670 plays a regulatory role in the mechanism through which cigarette smoke enhances the stemness and EMT of GES-1 cells. Furthermore, the Wnt/β-catenin pathway might be downstream of cigarette smoke-induced exosomal circ0000670 with respect to the promotion of gastric cancer progression. In conclusion, circ0000670 transported by gastric cancer cell exosomes regulates the Wnt/β-catenin pathway, affecting the biological characteristics of surrounding GES-1 cells and ultimately promoting the development of gastric cancer.

Cigarette smoke plays an important role in the occurrence and development of gastric cancer [42,43], but it is unclear whether cigarette smoke can promote the development of gastric cancer via exosomes. Exosomes are an important part of the communication system between cells and within organs, and they can transfer active molecules and biological signals from one cell type or tissue to another [6,7]. Many studies have indicated that exosomes play a decisive role in the occurrence and development of gastric cancer [8,9]. Cigarette smoke is involved in the occurrence and development of a variety of diseases by affecting exosomes [28,44,45]. In this study, we mainly clarified that cigarette smoke could affect the fate of surrounding GES-1 cells through gastric cancer cell exosomes and then promote the development of gastric cancer. Gastric cancer cell-derived exosomes were extracted before and after cigarette smoke exposure, and we examined the effects of cigarette smoke on exosomes. The results identified that there were no significant changes in exosome size and average secretion after cigarette smoke exposure. EMT is a common cellular process. Cells lose epithelial properties and acquire mesenchymal properties. Normal cells can acquire EMT properties, which may be an important feature in the carcinogenic process. Meanwhile, EMT participates in pro-cancerous tissue remodeling of gastric cancer and other tumors. Many cancers, including gastric cancer, arise from cancer stem cells. Therefore, the enhancement of stem cell characteristics plays an important indicator role in the occurrence and development of gastric cancer. We also observed the effects of cigarette smoke-treated gastric cancer cell exosomes on the stemness and EMT of GES-1 cells. We found that cigarette smoke-induced exosomes could promote the expression of Nanog, OCT4, Lin28 and vimentin and inhibit the expression of E-cadherin. In addition, the migration of GES-1 cells was also significantly enhanced (Figure 3). These results suggested that cigarette smoke-induced exosomes of gastric cancer cells promote the proliferation, stemness and EMT of GES-1 cells, and play an important role in gastric cancer progression.

Exosomes carry noncoding RNA, protein and other molecules to participate in cell communication and have a critical role in gastric cancer and other cancers [46,47]. The abnormal expression of circRNAs is closely related to the occurrence and development of gastric cancer [20,48,49]. It was found that cigarette smoke affects circRNAs and other noncoding RNAs transported by exosomes and plays a role in the occurrence and development of diseases [27,28,32]. In the current study, we investigated whether cigarette smoke promotes gastric cancer by affecting the components carried by exosomes, such as circRNA. We first screened and verified abnormally expressed circRNAs from the GEO database (GSE83521 and GSE93541) and tissues of gastric cancer patients with a smoking history. The results indicated that circ0000670 is highly expressed in the tissues of gastric cancer patients with a smoking history. Liu et al. also reported that circ0000670 plays an important role in the development of gastric cancer [50], but its role in cigarette smoke-induced gastric cancer and the effects of cigarette smoke-induced gastric cancer cell exosomes on the fate of surrounding normal cells was not clear. Our results showed an increasing trend in 40 gastric cancer patients with a smoking history (85%). We also found that cigarette smoke promoted the expression of circ0000670 in gastric cancer cells (HGC-27 and AGS) and associated exosomes (Figure 4). To study the role of exosomal circ0000670 in promoting the stemness and EMT of GES-1 cells, we commissioned a biological company to construct circ0000670 knockdown and overexpression lentivirus vectors. In further experiments, circ0000670 knockdown and overexpression lentivirus vectors were transferred into cigarette smoke-treated gastric cancer cells, and the expression of circ0000670 in gastric cancer cell exosomes was detected. We found that the augmenting effect of gastric cancer cell exosomes on the stemness and EMT of GES-1 cells was inhibited by the knockdown of circ0000670 (Figure 5C–G). In contrast, the results showed that gastric cancer cell exosomes of the circ0000670-overexpressing group could promote the stemness and EMT of GES-1 cells (Figure 5H–L).

In order to further explore the mechanism through which cigarette smoke-induced exosomal circ0000670 promotes the development of gastric cancer, we performed bioinformatic analysis and found that circ0000670 was associated with the Wnt/β-catenin signaling pathway. A previous study revealed that the activation of the Wnt/β-catenin pathway promotes the occurrence and development of cancers, including gastric cancer [51,52,53]. CircRNA also affects the occurrence and development of gastric cancer by regulating the activity of the Wnt/β-catenin pathway [21,54]. In this study, gastric cancer cells were treated with the β-catenin-targeting inhibitor jw55, cigarette smoke and lentivirus, alone or in combination, and then the exosomes were extracted. The results suggested that the Wnt/β-catenin pathway might be downstream of cigarette smoke-induced exosomal circ0000670 with respect to the effects on the surrounding normal cells and the promotion of gastric cancer progression.

## 5. Conclusions

In summary, cigarette smoke-induced gastric cancer cell exosomal circ0000670 acts as a promoter of gastric cancer by affecting the fate of surrounding normal cells and might be a potential target for the prevention and treatment of gastric cancer. These findings provide new insights into the mechanism of cigarette smoke-related gastric cancer.

## Figures and Tables

**Table 1:** Primer sequences.

Gene Name	Primer Sequences (5′-3′)
OCT4	F:5′-TGGAGAAGGTGGAACCAACT-3′
	R:5′-AGATGGTGGTCTGGCTGAAC-3′
NANOG	F:5′-GGAACGCCTCATCAATGC-3′
	R:5′-TGTCAGCCTCAGGACTTGAGA-3′
SOX2	F:5′-ACACCAATCCCATCCACACT-3′
	R:5′-GCAAACTTCCTGCAAAGCTC-3′
N-cadherin	F:5′-CTCCACTTCCACCTCCACAT-3′
	R:5′-GGACTCGCACCAGGAGTAAT-3′
E-cadherin	F:5′-GGACTCGCACCAGGAGTAAT-3′
	R:5′-TTGGCTGAGGATGGTGTAAG-3′
Vimentin	F:5′-GAGCTGCAGGAGCTGAATG-3′
	R:5′-AGGTCAAGACGTGCCAGAG-3′
circ0000670	F:5′-GGTTCATACCTCTAATTCATGTGG-3′
	R:5′-CATTTTCTTCCTAGACAAAGCCTTA-3′
β-catenin	F:5′-TGACACCTCCCAAGTCCTTT-3′
	R:5′-TTGCATACTGCCCGTCAAT-3′
GAPDH	F:5′-GCTGCCCAACGCACCGAATA-3′
	R:5′-GAGTCAACGGATTTGGTCGT-3′

## Data Availability

All data generated or analyzed in this study are included in thispublished article.
